# Proteasome Inhibition Contributed to the Cytotoxicity of Arenobufagin after Its Binding with Na, K-ATPase in Human Cervical Carcinoma HeLa Cells

**DOI:** 10.1371/journal.pone.0159034

**Published:** 2016-07-18

**Authors:** Qingxi Yue, Hong Zhen, Ming Huang, Xi Zheng, Lixing Feng, Baohong Jiang, Min Yang, Wanying Wu, Xuan Liu, Dean Guo

**Affiliations:** 1 Institute of Oncology, Shanghai 9th People's Hospital, Shanghai Jiao Tong University School of Medicine, Shanghai, 201999, P.R. China; 2 Shanghai Research Center for Modernization of Traditional Chinese Medicine, Shanghai Institute of Materia Medica, Shanghai Institutes for Biological Sciences, Chinese Academy of Sciences, Shanghai, 201203, P.R. China; 3 Center for Integrative Proteomics Research, BioMaPS Institute and Department of Chemistry and Chemical Biology, Rutgers, The State University of New Jersey, Piscataway, NJ, 08854–8076, United States of America; 4 Department of Chemical Biology, Ernest Mario School of Pharmacy, Rutgers, The State University of New Jersey, Piscataway, NJ, 08854–8020, United States of America; Universidade Federal do Rio de Janeiro, BRAZIL

## Abstract

Although the possibility of developing cardiac steroids/cardiac glycosides as novel cancer therapeutic agents has been recognized, the mechanism of their anticancer activity is still not clear enough. Toad venom extract containing bufadienolides, which belong to cardiac steroids, has actually long been used as traditional Chinese medicine in clinic for cancer therapy in China. The cytotoxicity of arenobufagin, a bufadienolide isolated from toad venom, on human cervical carcinoma HeLa cells was checked. And, the protein expression profile of control HeLa cells and HeLa cells treated with arenobufagin for 48 h was analyzed using two-dimensional electrophoresis, respectively. Differently expressed proteins in HeLa cells treated with arenobufagin were identified and the pathways related to these proteins were mapped from KEGG database. Computational molecular docking was performed to verify the binding of arenobufagin and Na, K-ATPase. The effects of arenobufagin on Na, K-ATPase activity and proteasome activity of HeLa cells were checked. The protein-protein interaction network between Na, K-ATPase and proteasome was constructed and the expression of possible intermediate proteins ataxin-1 and translationally-controlled tumor protein in HeLa cells treated with arenobufagin was then checked. Arenobufagin induced apoptosis and G2/M cell cycle arrest in HeLa cells. The cytotoxic effect of arenobufagin was associated with 25 differently expressed proteins including proteasome-related proteins, calcium ion binding-related proteins, oxidative stress-related proteins, metabolism-related enzymes and others. The results of computational molecular docking revealed that arenobufagin was bound in the cavity formed by the transmembrane alpha subunits of Na, K-ATPase, which blocked the pathway of extracellular Na^+^/K^+^ cation exchange and inhibited the function of ion exchange. Arenobufagin inhibited the activity of Na, K-ATPase and proteasome, decreased the expression of Na, K-ATPase α1 and α3 subunits and increased the expression of WEE1 in HeLa cells. Antibodies against Na, K-ATPase α1 and α3 subunits alone or combinated with arenobufagin also inhibited the activity of proteasome. Furthermore, the expression of the possible intermediate proteins ataxin-1 and translationally-controlled tumor protein was increased in HeLa cells treated with arenobufagin by flow cytometry analysis, respectively. These results indicated that arenobufagin might directly bind with Na, K-ATPase α1 and α3 subunits and the inhibitive effect of arenobufagin on proteasomal activity of HeLa cells might be related to its binding with Na, K-ATPase.

## Introduction

Cardiac steroids /Cardiac glycosides, which are the compounds used for treating cardiac failure, display strong anti-cancer activity to induce activation of cell death or impairment of cell proliferation by epidemiological data as well as *in vitro* and *in vivo* studies, and so it is possible to develop cardiac steroids /cardiac glycosides as anti-cancer agents. Promising compounds such as Anvirzel and UNBS1450 were in clinical trials in U.S.A and Belgium, respectively. A Phase I study of Anvirzel in patients with advanced solid tumours was approved by the US Food and Drug Administration (FDA) in 2000. Indeed, the completed phase I and phase II clinical trials with Anvirzel (a Nerium oleander extract containing several cardiac steroids but particularly enriched in oleandrin), either alone or more often in combination with other anticancer agents, had demonstrated acceptable safety profiles but limited efficacy in patients with solid tumors[1]. In 2006, UNBS1450, which was a semi-synthetic derivative of the novel cardenolide 2′′-oxovoruscharin (19-hydroxy-2”oxovoruscharin), entered Phase I clinical trials in Belgium. While preserving potent anti-proliferative properties patients with advanced solid tumors, minimal cardiotoxicity of UNBS1450 was found in clinical trials [2].

Cardiac steroids /Cardiac glycosides comprise mainly cardenolides with a five-membered unsaturated butyrolactone ring and bufadienolides with a six-membered unsaturated pyrone ring. Toad venom extracted from skins and postauricular glands of *Bufo bufo gargarizans Cantor* is called as “Chan-Su” in China, containing bufadienolides[3]. It has been widely used as an antimicrobial, anodyne, antineoplastic, cardiotonic, and local anesthetic agent for thousands of years. Toad venom is also the major component of several popular traditional Chinese medications such as Shexiangbaoxinwan, Liushenwan, and Niuhuangxiaoyanwan, which have long been used as alternative medicines in China, Japan, Korea, and other Asian countries [4]. Toad glandular secretions and skin extractions can be made to different types including oral solution, injection, ointment, and coating agent. One of the most widely used commercial preparation containing “Chan-su” is named Huachansu (Cinobufacini) injection, which is presently used for clinical cancer therapy in China[5]. A pilot study of Huachansu injection in patients with hepatocellular carcinoma (HCC), non-small-cell lung cancer (NSCLC), and pancreatic cancer showed that Huachansu injection improved the quality of life of patients and even enhanced tumor shrinkage with little toxicity[6]. Moreover, a case-control trial (*n* = 120) was conducted to assess the effects of Huachansu injection plus Jiedu granules (a Chinese medicine compound) *versus* transcatheter arterial chemoembolization (TACE) in post-surgical patients with HCC in Changhai Hospital (Shanghai, China). Huachansu injection plus Jiedu granules could postpone tumor recurrence and metastasis, prolong survival time and increase survival rate of post-surgical patients with HCC[7]. The meta-analysis demonstrated that cinobufacini combined with TACE could significantly increase the objective response rate and 2-year survival rate compared with TACE only in patients with advanced hepatocellular carcinoma[8]. A case of advanced lung cancer with malignant pericardial effusion treated by intrapericardial Huachansu injection instillation was reported[9]. Evidence from a meta-analysis showed Huachansu injection was beneficial in treating advanced non-small-cell lung cancer, combined with chemotherapy[10]. The clinical study using Huachansu injection in combination with gemcitabine and oxaliplatin in treating gallbladder carcinomas showed that Huachansu injection substantially enhanced the antitumor efficacy of gemcitabine and oxaliplatin and improved the quality of life of patients[11]. Compared with chemotherapy control group, Huchansu injection combined with chemotherapy provided benefits for advanced gastric cancer on increasing Karnofsky score, improving the response rate, reducing leucocytopenia and major side effects such as gastrointestinal side effects caused by chemotherapy[12].

Na, K-ATPase, also known as Na, K-pump, is a ubiquitously expressed transmembrane transporter in mammals, composed of tetramers of alpha and beta subunits[13]. The alpha subunit (a large polypeptide of about 1,000 amino acid residues) catalyzes the ion-dependent ATPase activity and carries the binding sites for ATP and the specific inhibitor ouabain and the beta subunit (a smaller polypeptide of about 300 residues) regulates conformational stability and activity of the alpha subunit. Additionally, the complex of alpha and beta subunits in Na, K-ATPase associates with members of the FXYD proteins. The normal activity of Na, K-pump is essential for maintaining ionic homeostasis, physiological electrochemical gradient, cellular pH, and cell volume[14]. Na, K-ATPase is critical in maintaining high extracellular Na (~145 mM) and high intracellular K (~150 mM) by pumping Na ions out of the cell and importing K ions into the cell. The major signaling pathways activated by Na,K-ATPase were reported including p21 Cip, the epithelial-to-mesenchymal transition (EMT), phosphoinositide 3-kinase (PI3K)/protein kinase B (Akt)/the mammalian target of rapamycin (mTOR) pathway, p38 mitogen-activated protein kinase (MAPK) cascade, and cholesterol homeostasis[15]. Na, K-ATPase is also highly expressed in cancer cells and the activity of Na, K-pump increases during the course of malignant cell transformation, which indicate that Na, K-ATPase may serve as a biological marker and a therapeutic target of cancer cells[16]. The anti-cancer effects of cardiac steroids/cardiac glycosides were reported to be related to physiological events such as alterations in gene-expression profiles, membrane fluidity, N-linked glycan expression and the homeostasis of K(+), Na(+) and Ca(2+), inhibition of glycolysis, topoisomerase II and TNF/NF-κB pathway, upregulation of DR4 and DR5, increased the level of p21, increased the production of reactive oxygen species, and others[17]. While, the anti-cancer mechanism of cardiac steroids /cardiac glycosides was not clear enough. Firstly, whether the downsteam signal cascades of cardiac steroids/cardiac glycosides are all related to their binding with Na, K-ATPase was still a subject of ongoing research. Secondly, previous studies suggested that Na, K-ATPase could function as a versatile signal transducer except as an energy transducing ion pump[15,17]. As reported before, binding of cardiac steroids/cardiac glycosides to Na, K-ATPase might activate multiple downstream signal transduction pathways [1–2,15,17–20]. The signal cascades after Na, K-ATPase bound by cardiac steroids/cardiac glycosides to are so complicated and far from clear now.

Arenobufagin, a representative natural bufadienolide compound, is the major active component extracted from toad venom. Cruz Jdos S et al reported arenobufagin blocked the Na+/K+ pump current of dissociated guinea-pig cardiac myocytes in a dose-dependent manner[21]. Cruz Jdos S et al also reported arenobufagin, as a potent Na+/K+ pump inhibitor, depressed the delayed rectifier K+ current of single guinea-pig ventricular cells in the whole-cell patch-clamp configuration[22]. Perera Córdova WH et al reported arenobufagin fully inhibited the Na, K-ATPase activity of normal human renal tissues in a concentration-dependent manner[23]. Li M et al reported arenobufagin inhibited vascular endothelial growth factor (VEGF)-mediated angiogenesis through suppression of VEGFR-2-mediated signaling cascades[24]. Li M et al reported arenobufagin induced apoptosis and autophagy in HCC cells through inhibition of phosphatidylinositol 3-kinase (PI3K)/Akt/mammalian target of rapamycin (mTOR) signal pathway[25]. Deng LJ et al reported arenobufagin directly intercalated with DNA, leading to double-strand DNA breaks and triggering DNA damage response *via* the Ataxia telangiectasia mutated (ATM) /Ataxia telangiectasia and Rad3 related(ATR) signaling pathway, which subsequently resulted in G2 phase cell cycle arrest of HCC cells[26].

In the present study, the cytotoxicity of arenobufagin on human cervical carcinoma HeLa cells was firstly examined. Then, a proteomic technology was used to identify differently expressed proteins in HeLa cells treated with arenobufagin for 48 h. 2-DE was conducted and then differentially expressed proteins were identified by MALDI-TOF MS/MS. A comprehensive network analysis was implemented to mine the functional association between the experimentally defined proteins and the pathways related to the possible target-related proteins of arenobufagin were also mapped. The possible intermediate protein partners between Na, K-ATPase and proteasome-related proteins in HeLa cells treated with arenobufagin were then predicted by using protein-protein interaction network. Computational molecular docking was performed to verify the binding of arenobufagin and Na, K-ATPase. Finally, the effects of arenobufagin on the activity of Na, K-ATPase and cellular proteasome in HeLa cells were examined. The expression of the possible intermediate proteins ataxin-1 and translationally-controlled tumor protein in HeLa cells treated with arenobufagin was also checked, respectively.

## Materials and Methods

### Materials and reagents

**Fig 1:**
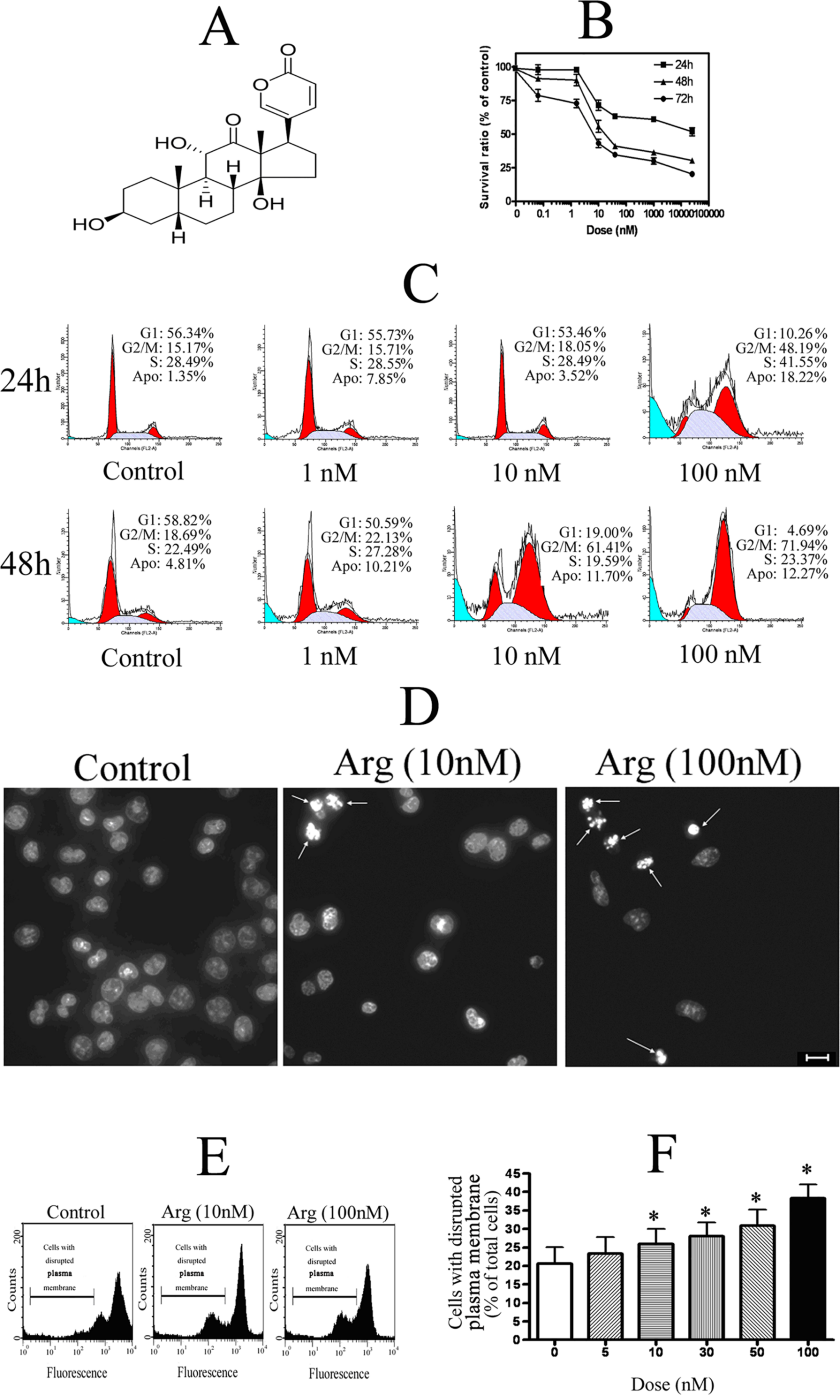
Cytotoxicity of arenobufagin (Arg) on HeLa cells. (**A**) Chemical structure of arenobufagin. (**B**) Cell viability of HeLa cells treated with arenobufagin at different doses for 24 h, 48 h and 72 h. (**C**) Representative DNA histograms of HeLa cells obtained by flow cytometry analysis. Accumulation in G_2_/M phase was observed in HeLa cells treated with 100 nM arenobufagin for 24 h or 10 nM, 100 nM arenobufagin for 48 h. (**D**) Morphological change induced by 10 nM or 100 nM arenobufagin in HeLa cells—for 48 h (scale bar = 10 μm). Typical change of apoptotic morphology in arenobufagin-treated cells was observed. (**E**) Representative flow cytometry result of plasma membrane potential by rhodamine staining assay. Treated with arenobufagin for 3 h, HeLa cells with the potential of disrupted plasma membrane were count. (**F**) Quantitation in percentage of HeLa cells with the potential of disrupted plasma membrane. Data were statistical results of three independent experiments. Data were expressed as mean ± SD. *Significant difference from the control group at P<0.05.

Arenobufagin with a purity of more than 98% was isolated and purified from Chan Su by laboratory of TCM chemistry, Shanghai Research Center for Modernization of Traditional Chinese Medicine, Shanghai Institute of Materia Medica, Chinese Academy of Sciences as reported before[27]. The chemical structure of arenobufagin was shown in Fig 1A. All reagents used in 2-dimensional electrophoresis (2-DE) were purchased from Bio-Rad Laboratories (Hercules, CA, USA). Other chemical reagents, except where specially noted, were purchased from Sigma-Aldrich Chemical Co. (St. Louis, MO, USA).

### Cell culture

The human cervical carcinoma HeLa cell line (CCL-2) were obtained from the American Type Culture Collection (ATCC, Rockville, MD, USA). HeLa cells were cultured in minimum essential medium (Life Technologies, Gaithersburg, MD, USA) with 2 mM L-glutamine, 2.2 g/l sodium bicarbonate, 0.1 mM non-essential amino acids, and 1.0 mM sodium pyruvate plus 10% fetal bovine serum (FBS). Antibiotics added were 100 units/ml penicillin and 100 μg/ml streptomycin (Invitrogen, Karlsruhe, Germany) in complete medium.

### MTT assay

The effect of arenobufagin on cell viability was determined by MTT assay as reported before[28]. Briefly, HeLa cells were plated in 96-well flat-bottomed plates (Corning Incorporated, Acton, MA, USA) at density of 1×10^3^ cells/well in complete medium and incubated overnight. Then, the medium was changed into fresh medium containing various amounts of arenobufagin for 24h, 48h or 72h. At the end of the incubation, 20 μl of MTT (5 mg/ml) was added to each well and the plates were incubated for 3 h at 37°C. Then, 100 μl of lysis buffer (20% sodium dodecyl sulfate [SDS] in 50% N,N-dimethylformamide, containing 0.5% [v:v] 80% acetic acid and 0.4% [v:v] 1N HCL) was added to each well and incubated overnight (16 h). Cell viability was evaluated by measuring the mitochondrial-dependent conversion of the yellow tetrazolium salt MTT to purple formazan crystals by metabolic active HeLa cells. The optical density (proportional to the number of live cells) was assessed with a Microplate Reader Bio-Rad 550 at 570 nm. Each experiment was performed in triplicate. Results of three independent experiments were used for statistical analysis. IC_50_ value (half-maximal inhibitory concentration) was calculated by the Logit method.

### Flow cytometric analysis of cell cycle

Flow cytometric analysis of cell cycle was conducted as reported before[28]. Briefly, adherent and detached HeLa cells were harvested with trypsin, washed with PBS for 3 times and then fixed in ice-cold 70% ethanol at 4°C for 2 h. After centrifugation at 100×g for 2 min, HeLa cells were resuspended in propidium iodide stain buffer (0.1% Triton X-100, 10 μg/mL DNase-free RNase A, 50 μg/mL PI in PBS) for 30 min in dark. Flow cytometric analysis was conducted using a Becton Dickson FACStar Plus Flow Cytometer.

### Fluorescent staining of cell nuclei

After treated with arenobufagin, HeLa cells were washed twice with PBS, fixed with 4% paraformaldehyde (pH 7.4) for 30 min at room temperature and then stained with the DNA-specific dye Hoechst 33258 (0.05 mg/ml). The cells were incubated with the dye at 37°C for 10 min and again washed in PBS. The images of cell nuclei staining were observed using an Olympus UV light fluorescence microscope.

### Measurement of plasma membrane potential

Plasma membrane potential was determined by rhodamine 123, a cell-permeable cationic dye that preferentially entered into the plasma based on highly negative plasma membrane potential. Depolarization of plasma membrane potential resulted in the loss of Rhodamine 123 from the mitochondria and a decrease in intracellular fluorescence. Briefly, after treated with arenobufagin, HeLa cells were trypsinized, washed twice with PBS, and then resuspended in 500 μl of culture medium containing 5 μM rhodamine 123 to incubate for 30 min at 37°C in the dark. The cells were then rinsed with PBS and resuspended in 500 μl of PBS for flow cytometric analysis.

### Proteomic analysis (2-DE combined with MS/MS identification)

The proteomic analysis was conducted similar to our previous studies[28–32]. Briefly, HeLa cells treated with arenobufagin (0.1 nM, similar to its IC_50_ value) or solvent control (0.1% DMSO) were harvested with trypsin. After washed with PBS, cell pellets were dissolved in lysis buffer containing 7 M urea, 2 M thiourea, 2% CHAPS, 1% DTT, 0.8% Pharmalyte and protease-inhibitor. Homogenization of the cells was achieved by ultrasonication (10 strokes, low amplitude) on ice. The lysed cells were centrifuged at 15000 × g for 30 min at 4°C and the supernatant containing the solubilized proteins was used directly or stored at -80°C. 2-DE was carried out using Bio-Rad 2-DE system. Briefly, 150 μg protein sample was applied for isoelectric focusing (the first dimensional electrophoresis) using the ReadyStrip IPG Strips (17 cm, pH 4–7 Bio-Rad). The strips were rehydrated at 50 V for 12h in a Protean IEF cell (Bio-Rad) and then the proteins were separated, based on their pI, according to the following protocol: 250 V with linear climb for 30 min, 1000 V with rapid climb for 60 min, 10000 V with linear climb for 5 h and 10000 V with rapid climb until 60 000 Vh was reached. After isoelectric focusing, IPG strips were equilibrated with Equilibration Buffer I (containing 50 mM Tris-HCL, pH 8.8, 30% glycerol; 7 M urea, 2% SDS and 1% DTT) and Buffer II (similar to Buffer I but containing 4% iodoacetamide instead of DTT) for 15 min each. The IPG strips were then directly applied to 12% homogeneous SDS-PAGE gels for electrophoresis using a PROTEIN II xi Cell system (Bio-Rad). Silver Stain Plus kit (Bio-Rad) was used to detect protein spots according to the manufacturer's instructions. The silver-stained gels were then scanned using a Densitometer GS-800 (Bio-Rad) and then analyzed using PD-Quest software (Bio-Rad). Paired (control and arenobufagin-treated) protein samples from 3 independent experiments were analyzed by 2-DE. And, for each pair of protein samples, quadruplicate electrophoreses were performed to ensure reproducibility. Totally 24 gels (12 pairs) were used in the present study. The individual protein spot quantity was normalized (the raw quantity of each spot in a member gel was divided by the total density of the gel) and the normalized spot intensities were expressed in ppm. The method of Statistical analysis to find proteins with significantly differentially-expressed level in arenobufagin-treated group compared with control group was conducted as described below in “Statistical analysis”. These differentially-expressed protein spots were cut from the gels and used for further identification by MALDI-TOF MS/MS on an ABI 4700 Proteomics Analyzer with delayed ion extraction (Applied Biosystems). Briefly, gel pieces were destained, washed, shrunk by dehydration and then digested with trypsin. Extracted peptides were mixed with 0.7 μl MALDI matrix (5 mg/ml CHCA diluted in 0.1% TFA/50% ACN) and spotted on to the 192-well stainless steel MALDI target plates. Mass spectra were obtained in a mass range of 700–3200 Da, using a laser (355 nm, 200 Hz) as desorption ionization source. Using the individual PMF spectra, peptides exceeding a signal-to-noise ratio of 20 that passed through a mass exclusion filter were submitted to fragmentation analysis. The parameters for peak matching included Min S/N: 20 and Mass Tolerance: 0.2 Da. Min Peak to Match reference masses was 4 and Max outlier Error was set to 100 ppm. Total shots for each MS spectrum were 2000 while total shots for each MS/MS spectrum were 3000. MS/MS accuracy was calibrated against the MS/MS fragments of m/z 1606.85, which was one of the peaks generated in myoglobin PMF. Peptide differential modifications allowed during the search were carbamidomethylation of cysteines and oxidation of methionines. The maximum number of missed cleavages was set to 1 with trypsin as the protease. The database search was performed by using the MASCOT search engine (Matrix Science, London, United Kingdom) to screen the NCBI protein sequence database restricted to human taxonomy. Protein homology identifications of the top hit (first rank) with a relative score exceeding 95% probability and additional hits (second rank or more) with a relative score exceeding 98% probability threshold were retained. The probability-based score, assuming that the observed match was significant (*P*<0.05), had to be more than 64 when submitting PMF data to the database, and be more than 30 for individual peptide ions when submitting peptide sequence spectra. Proteins belonging to a protein family with multiple members were singled out based on the identification of unique and diagnostic peptides.

### Map pathways related to possible target-related proteins of arenobufagin

The possible target-related proteins detected in proteomic study above were first mapped onto the KEGG pathway database. Then, the obtained pathways and the proteins were organized to generate a bipartite graph of protein-pathway association, in which a protein and a pathway were linked if the protein appeared in this pathway.

### Molecular docking of arenobufagin and Na, K-ATPase

Molecular docking simulation was performed to investigate detailed binding modes between arenobufagin and Na, K-ATPase. The E2P crystal structure of pig kidney Na, K-ATPase α_1_β_1_γ (PDB ID: 4RES) was deployed for molecule docking simulation. Sequence identity between human alpha subunits (α_1,_ α_2,_ α_3_ and α_4_) and α_1_ of other species, whose crystal structures (PDB ID: 4RES, 4HYT and 4XE5) were available in the PDB database, were calculated with the SWISS-MODEL[33]. The ligand, arenobufagin, was optimized by using the semi-empirical PM6 method as implemented in the Gaussian 09. The software package AutoDock 4.2 was used for the molecular docking calculation with default parameters [34].

### Assay of Na, K-ATPase activity

The activity of Na, K-ATPase was assessed as reported before[35, 36]. According to the kit protocol (Nanjing Jiancheng Bioengineering Institute, China), Na, K-ATPase activity was assayed by measuring the release of inorganic phosphate (Pi) from ATP hydrolysis. Briefly, HeLa cells, treated with either solvent control (0.1% DMSO) and arenobufagin at different concentrations, were cultured in minimum essential medium (Life Technologies, Gaithersburg, MD, USA) with 1.8 mM CaCl_2_, 0.8 mM MgSO_4_, 5.4 mM KCl, 116.4 mM NaCl,1.0 mM NaH_2_PO_4_, 26.2 mM NaHCO_3_, 1.0 mM sodium pyruvate, 2 mM L-glutamine and 0.1 mM non-essential amino acids plus 10% fetal bovine serum (FBS) and antibiotics (100 units/ml penicillin and 100 μg/ml streptomycin) for 24h and then lysed with normal saline for 30min on ice. Lysates were then centrifuged at 12,000×g for 15min and the homogenates were centrifuged to obtain the supernatant. Protein concentration was determined by the kit of Bradford protein assay (Bio-Rad, USA). Samples (2 μg protein) were suspended in 70 μL assay buffer (10 mmol L^-1^ KCl, 5 mmol L^-1^ MgCl_2_, 40 mmol L^-1^ NaCl, 50 mmol L^-1^ Tris-base, 5 mmol L^-1^ EGTA, pH 7.4). After 30 min, the reaction was terminated by adding 1 mL Biomol Green reagent at room temperature. Finally, the activity of Na, K-ATPase was assessed by measuring the amount of inorganic phosphate with malachite green dye method and then expressed as micromoles per milligram protein. Released inorganic phosphate (Pi) was detected using a malachite-based Biomol Green reagent.

### Flow cytometry analysis of intracellular Ca(2+) and ROS levels

HeLa cells treated with arenobufagin or solvent control were harvested with trypsin. After PBS washes, cells were incubated with 5 μM Fluo-3-AM (for measurement of the Ca2+) or 100 μM 2’7’-dichlorodihydrofluorescein diacetate (for measurement of the ROS level) for 10 min. After washes with PBS, fluorescence was measured using the Becton Dickson FACStar Plus Flow Cytometer. Data were collected and the mean fluorescence intensity of at least 10, 000 of cells was analyzed by Cellquest software (version 3.2).

### Effect of ROS scavenger on the cytotoxicity of arenobufagin

The effect of ROS scavenger, N-acetyl cysteine, on the cytotoxicity of arenobufagin was checked using the method similar to our previous report [29]. Briefly, HeLa cells were preincubated with N-acetyl cysteine (1 mM, 10 mM or 20 mM) for 2 h followed by treatment with arenobufagin for 72 h. The viability of HeLa cells was then determined using MTT method as described above.

### Measurement of cytosolic proteasomal activity

The enzymatic activity of ubiquitin C-terminal hydrolase in cell lysate was measured similar to previous reports [37, 38]. Briefly, HeLa cells were treated with arenobufagin at different doses (1 nM, 10 nM, 100 nM, 1000 nM, 10000 nM), solvent control (0.1% DMSO), antibody against α1 subunit of Na,K-ATPase (20 μg/ml, mouse monoclonal antibody, Santa Cruz Biotechnology, sc-514614, U.S.A), antibody against α3 subunit of Na,K-ATPase (20 μg/ml, mouse monoclonal antibody, Santa Cruz Biotechnology, sc-376967, U.S.A), a control unrelated antibody (20 μg/ml, mouse anti-actin monoclonal antibody, Sigma) alone or combinated with 10nM arenobufagin for 24h, 48h and 72h, respectively. After treatment, HeLa cells were washed with PBS and then lysed with a lysis buffer (50 mM Tris-HCl, pH 8.0, 0.5 mM EDTA, 150 mM NaCl, 0.5% Nonidet P-40, 0.5 mM phenylmethylsulfonyl fluoride and 0.5 mM dithiothreitol) for 30 min at 4°C. The homogenate was then centrifuged at 15000 × g for 30 min at 4°C. The supernatant was collected as the whole cell extract and the protein content in the supernatant was measured with the Bradford reagent. Protease activity was assayed by adding the whole cell extract (containing about 50 μg protein) to 100 μl assay buffer (20 mM Tris-HCl, pH 8.0, 1 mM adenosine triphosphate, 2 mM MgCl_2_) containing 12.5 μM ubiquitin-AMC (Thermo Fisher Scientific, Rockford, IL 61105, U.S.A). By specifically cleaving the bond between the C-terminus of ubiquitin and AMC, the fluorogenic AMC component from ubiquitin-AMC could be released by ubiquitin C-terminal hydrolase. The assay samples were incubated for 30 min at 37°C, and subjected to fluorescence detection using a Microplate Reader Bio-Rad 550 with excitation wavelength and emission wavelengths of 360 and 480 nm, respectively.

Besides the above method, the enzymatic activities of cellular proteasome in cell lysate were also measured as reported in our previous paper [39, 40]. Briefly, HeLa cells were treated with solvent control (0.1% DMSO) or arenobufagin at different concentrations for 24 h at 37°C and then harvested, washed with PBS and then lysed in proteasome activity assay buffer for 30min at 4°C. The homogenate was then centrifuged at 12000 × g at 4°C for 30min. The supernatant was collected as whole cell extract and the protein content in the supernatant was measured with the Bradford reagent. Catalytic activities of cellular proteasome were assayed by adding whole cell extract (containing 30 μg protein) to 100 μl of assay buffer (20 mM Tris-HCl, pH 8.0, 2 mM MgCl_2_, 1 mM adenosine triphosphate) containing 50 μM fluorogenic peptide substrates (Thermo Fisher Scientific, Rockford, IL, U.S.A) such as Z-LLE-AMC (7-amido-4-methyl-coumarin) for detecting C-L activity(β1 subunit), Z-ARR-AMC for detecting T-L activity(β2 subunit) or Suc-LLVY-AMC for detecting CT-L activity(β5 subunit), respectively. The hydrolase activity of the catalytic subunits of proteasome (β1, β2, β5) could release the fluorogenic AMC component from the peptide substrates and the AMC release was measured after 30 minute incubation using a Microplate Reader Bio-Rad 550 with excitation and emission wavelengths of 360 and 480 nm, respectively.

### Western blotting analysis

HeLa cells (control or arenobufagin-treated) were washed three times with cold TBS, harvested using a cell scraper, and lysed in 10 volume of cold lysis buffer (50 mM Tris-HCl, pH 7.2, 250 mM NaCl, 0.1% NP-40, 2 mM EDTA, 10% glycerol, 1 mM PMSF, 5 μg/ml Aprotinin, 5 μg/ml Leupeptin) on ice. After centrifugation at 15000 × g for 30 min at 4°C, the supernatant protein was denatured by mixing with equal volume of 2 × sample loading buffer and then boiled at 100°C for 5 min. Briefly, an aliquot (50 μg) of the extracted protein was loaded onto a 12% SDS gel, separated electrophoretically, and transferred to a PVDF membrane (Bio-Rad). After blocking nonspecific protein with 10% dehydrated skim milk, the membrane was incubated with primary antibodies overnight at 4°C. The primary antibodies used in the present study were mouse anti-Na, K-ATPase α1 monoclonal antibody (1:200, Santa Cruz Biotechnology), mouse anti-Na, K-ATPase α3 monoclonal antibody (1:200, Santa Cruz Biotechnology), mouse anti-WEE1 monoclonal antibody (1:500, Abnova, Taipei City, Taiwan), mouse anti-GAPDH monoclonal antibody(1:2000, Sigma) and mouse anti-actin monoclonal antibody (1:2000, Sigma). Blots were then incubated with HRP-conjugated goat anti-mouse IgG (1:2000, Sigma) for 1 h at room temperature and then visualized using the chemiluminescence (Pierce Biotechnology, Rockford, IL, USA).

### Protein-protein interaction network construction

The signal cascade from Na, K-ATPase to proteasome had not been reported before. To predict the possible signal cascade between Na, K-ATPase and the proteasome-related proteins found in the proteomic study, protein-protein interaction network was constructed based on various online databases containing experimental information of protein interactions and associations as reported before[28]. The direct partners interacting with subunits of Na, K-ATPase or the proteasome-related proteins were further used as a new query seed to fish out another round of partner proteins. Through this way, the network was expanded step by step until the subunits of Na, K-ATPase and the proteasome-related proteins were connected.

### Flow cytometry analysis of ataxin-1 and translationally-controlled tumor protein levels

The levels of ataxin-1 and translationally-controlled tumor protein analyzed by flow cytometry were conducted as our previous report[41]. Briefly, after treated with arenobufagin at different doses, HeLa cells were collected, washed with PBS and fixed with 2% paraformaldehyde for 20 min at room temperature. After incubated with a solution (containing 0.1% TritonX-100 and 1% bovine serum album) overnight at 4°C to permeabilize cell membranes and block nonspecific protein binding, HeLa cells were then incubated for 60 min at room temperature with the primary antibodies, i.e. goat anti-ataxin-1 polyclonal antibody (Santa Cruz Biotechnology) and goat anti-(translationally-controlled tumor protein) polyclonal antibody (Santa Cruz Biotechnology) at the dilution of 1:200. After washed by PBS for three times, the cells were then incubated with the FITC-conjugated mouse anti-goat secondary antibody (1: 200, Santa Cruz Biotechnology) for 30 min at room temperature in dark. After washed by PBS for three times, the antigen density was measured using a Becton Dickson FACStar Plus Flow Cytometer.

### Statistical analysis

Data were given as the mean ± SEM. In proteomic assay, the normalized values of the densities of the protein spots were used for statistical analysis performed by the non-paired Student’s t-test assay in PD-Quest software (Bio-Rad). Comparison was made between gel images of arenobufagin-treated group (12 gels) and gel images of control group (12 gels). Significantly differentially-expressed spots were considered according to the criteria: two fold or more increased or decreased intensity between control group and arenobufagin-treated group as well as p<0.05. For the experiments other than proteomic assay, the statistical analysis was performed using the non-paired Student’s t-test in GraphPad Prism software (version 4.0, GraphPad Software, San Diego, CA). p<0.05 was considered as significantly different.

## Results

### Cytotoxicity of arenobufagin

Similar to bufalin, arenobufagin is one of the main components of the toad venom extract clinically used in China, according to our previous study[27]. As shown in Fig 1, arenobufagin exhibited the cytotoxicity against HeLa cells. Arenobufagin dose-dependently and time-dependently decreased the viability of HeLa cells by MTT assay (Fig 1B). Arenobufagin caused G2/M phase arrest and apoptosis of HeLa cells. The representative DNA histograms of HeLa cells exposed to arenobufagin by flow cytometry assay showed that 100 nM arenobufagin induced G_2_/M phase and apoptosis at 24 h while 10 nM arenobufagin and 100 nM arenobufagin could both induce G2/M phase and apoptosis at 48 h (Fig 1C). Typical morphological change of apoptosis could be found in HeLa cells treated with 10 nM or 100 nM arenobufagin for 48h by Hoechst staining (Fig 1D). Furthermore, plasma dysfunction of HeLa cells was involved in arenobufagin-induced apoptosis by rhodamine 123 staining. As shown in Fig 1E, the plasma membrane potential of HeLa cells could be decreased by arenobufagin. Arenobufagin dose-dependently increased the percentage of HeLa cells with disrupted plasma membrane potential (Fig 1F).

### Identification of differentially-expressed proteins treated with arenobufagin

**Fig 2:**
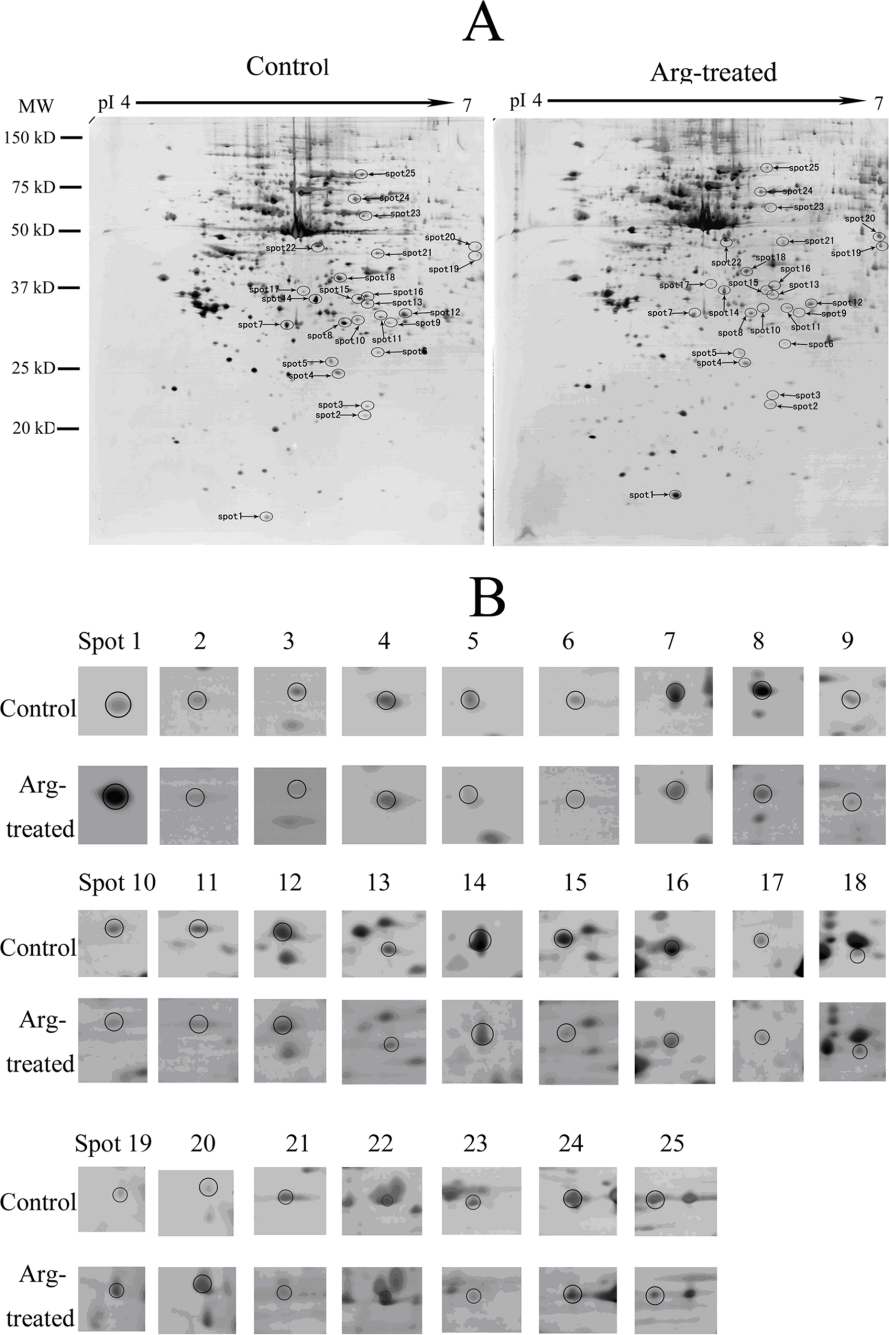
The proteome maps of control and arenobufagin (Arg)-treated HeLa cells. (**A**) Representative two-dimensional electrophoresis gel images of HeLa cells treated with 10 nM arenobufagin or solvent control for 48 h. Differentially-expressed protein spots were shown by the arrows. (**B**) The expanded region of differentially-expressed protein spots in A. The spots within the circles were differentially-expressed proteins.

Representative proteome maps (2-DE images) of 10 nM arenobufagin-treated HeLa cells were shown in Fig 2. The gel group (shown in Fig 2A) was the representative gel group of twelve replicate gel groups from three independent experiments. Differentially-expressed protein spots were shown by the arrows. The expanded regions of differentially expressed protein spots were shown in Fig 2B. The proteins within the circles were the differentially expressed proteins. By comparing the proteome maps of control HeLa cells and arenobufagin-treated HeLa cells with PDQUEST software, 25 significantly differentially expressed protein were found. The average intensity values, the standard deviation, the statistical assay results and the fold difference of the differentially expressed protein spots between control group and arenobufagin-treated group were shown in Table 1. The fold difference was represented by the ratio of the intensity value of arenobufagin-treated group to the intensity value of control group. MS/MS identification results of these differentially expressed proteins spots were summarized in Table 2. The protein score, coverage, number of identified peptides and best ion score of each protein spot were also shown in Table 2. In brief, 25 significantly differentially expressed protein included proteasome-related proteins (PSMB7, PSME1, PSME2, PSMD13, UBQLN2, UBE2K),calcium ion binding-related proteins (S100A6, PRKCSH), oxidative stress-related proteins(PRDX4,HSPB1, AHSA1), metabolism-related enzymes(SOD1, HIBADH, SRM, PPA1, AKR1A1, ACAT2, LDHB, PDIA3, RNPEP, ECHS1) and others(STMN1, TPM3, EEF1D, RANBP1) in the present study.

**Table 1:** Summary of differentially-expressed proteins in arenobufagin-treated HeLa cells compared with control.

Spot	Spot volume (ppm)		Fold difference	p value
	Control (Mean ± SEM)	arenobufagin–treated (Mean ± SEM)		
1	139.43 ± 43.76	713.77 ± 230.51	5.12	0.026
2	185.78 ± 45.07	61.34 ± 14.75	0.33	0.016
3	320.11 ± 2.05	132.09 ± 49.57	0.41	0.047
4	881.09 ± 273.08	277.29 ± 79.67	0.31	0.048
5	142.20 ± 53.22	28.20 ± 6.16	0.20	0.049
6	204.30 ± 43.64	65.58 ± 8.49	0.32	0.0059
7	1841.87 ± 327.73	901.40 ± 143.89	0.49	0.016
8	1887.71 ± 294.60	892.12 ± 203.73	0.47	0.012
9	162.61 ± 27.72	74.73 ± 13.63	0.46	0.010
10	675.76 ± 192.00	249.76 ± 37.44	0.37	0.040
11	762.49 ± 108.87	376.66 ± 64.05	0.49	0.0068
12	1641.62 ± 288.58	760.27 ± 189.63	0.46	0.021
13	984.94 ± 178.12	423.86 ± 99.29	0.43	0.012
14	2531.82 ± 373.57	1213.40 ± 157.13	0.48	0.0044
15	1416.89 ± 252.06	478.12 ± 93.79	0.34	0.0021
16	1403.52 ± 190.78	592.85 ± 93.15	0.42	0.00094
17	115.00 ± 19.71	45.21 ± 8.73	0.39	0.0046
18	1530.99 ± 155.17	748.14 ± 112.27	0.49	0.00086
19	156.90 ± 46.64	342.54 ± 68.29	2.18	0.035
20	231.03 ± 28.99	470.66 ± 67.87	2.04	0.0051
21	675.81 ± 89.25	320.98 ± 74.11	0.47	0.0062
22	1727.97 ± 251.46	849.79 ± 124.07	0.49	0.0064
23	946.62 ± 267.13	363.17 ± 82.49	0.38	0.049
24	1079.27 ± 200.30	532.26 ± 96.48	0.49	0.026
25	601.83 ± 86.04	244.40 ± 67.79	0.41	0.0049

**Table 2:** The results of differentially-expressed proteins identified using MALDI-TOF MS/MS.

Spot	Target protein		NCBI Accession Number	Theoretical molecular mass (kDa)/ p*I*	Protein score	Sequence coverage (%)	Best ion score
	full name	abbreviation					
1	S100 calcium-binding protein A6	S100A6	20664042	10.2/5.33	190	52	79
2	stathmin 1	STMN1	5031851	17.3/5.76	237	64	77
3	tropomyosin 3 isoform 2	TPM3	24119203	28.8/4.68	143	28	32
4	protein kinase C substrate, 60.1 kDa protein, heavy chain	PRKCSH	15488917	45.0/4.27	75	18	55
5	ubiquilin 2	UBQLN2	16753207	65.7/5.15	64	15	40
6	eukaryotic translation elongation factor 1 delta isoform 2	EEF1D	25453472	31.1/4.90	126	20	58
7	Ran-binding protein 1	RANBP1	938026	23.2/5.19	85	28	42
8	heat shock protein 27	HSPB1	662841	22.3/7.83	227	29	70
9	Cu/Zn-superoxide dismutase	SOD1	1237406	15.9/5.86	67	19	57
10	peroxiredoxin-4	PRDX4	49456297	30.6/5.86	241	39	97
11	proteasome beta 7 subunit protein	PSMB7	4506203	30.0/7.57	68	18	33
12	enoyl Coenzyme A hydratase,short chain,1, mitochondrial	ECHS1	14286220	31.4/8.34	125	20	95
13	proteasome activator subunit 1 isoform 1	PSME1	49456277	28.7/5.78	73	36	38
14	proteasome activator complex subunit 2	PSME2	18203506	27.0/5.44	165	25	47
15	3-hydroxyisobutyrate dehydrogenase	HIBADH	23308751	35.3/8.38	92	13	40
16	proteasome 26S non-ATPase subunit 13 isoform 1	PSMD13	28872728	42.9/5.53	108	16	49
17	spermidine synthase	SRM	134811	33.8/5.30	138	32	56
18	pyrophosphatase 1	PPA1	11056044	32.6/5.54	189	16	32
19	aldehyde reductase	AKR1A1	5174391	36.5/6.32	207	29	37
20	acetyl-CoA transferase-like protein	ACAT2	19880019	41.2/6.27	66	13	47
21	lactate dehydrogenase B	LDHB	4557032	36.6/5.71	190	32	31
22	activator of heat shock 90kDa protein ATPase homolog 1	AHSA1	6912280	38.2/5.41	135	27	53
23	ubiquitin-conjugating enzyme E2-25 kDa	UBE2K	60594411	22.5/5.33	87	49	30
24	protein disulfide isomerase	PDIA3	860986	56.6/6.10	95	20	35
25	aminopeptidase B	RNPEP	40316915	72.5/5.51	90	18	45

### Pathways related to the cytotoxicity of arenobufagin

**Fig 3:**
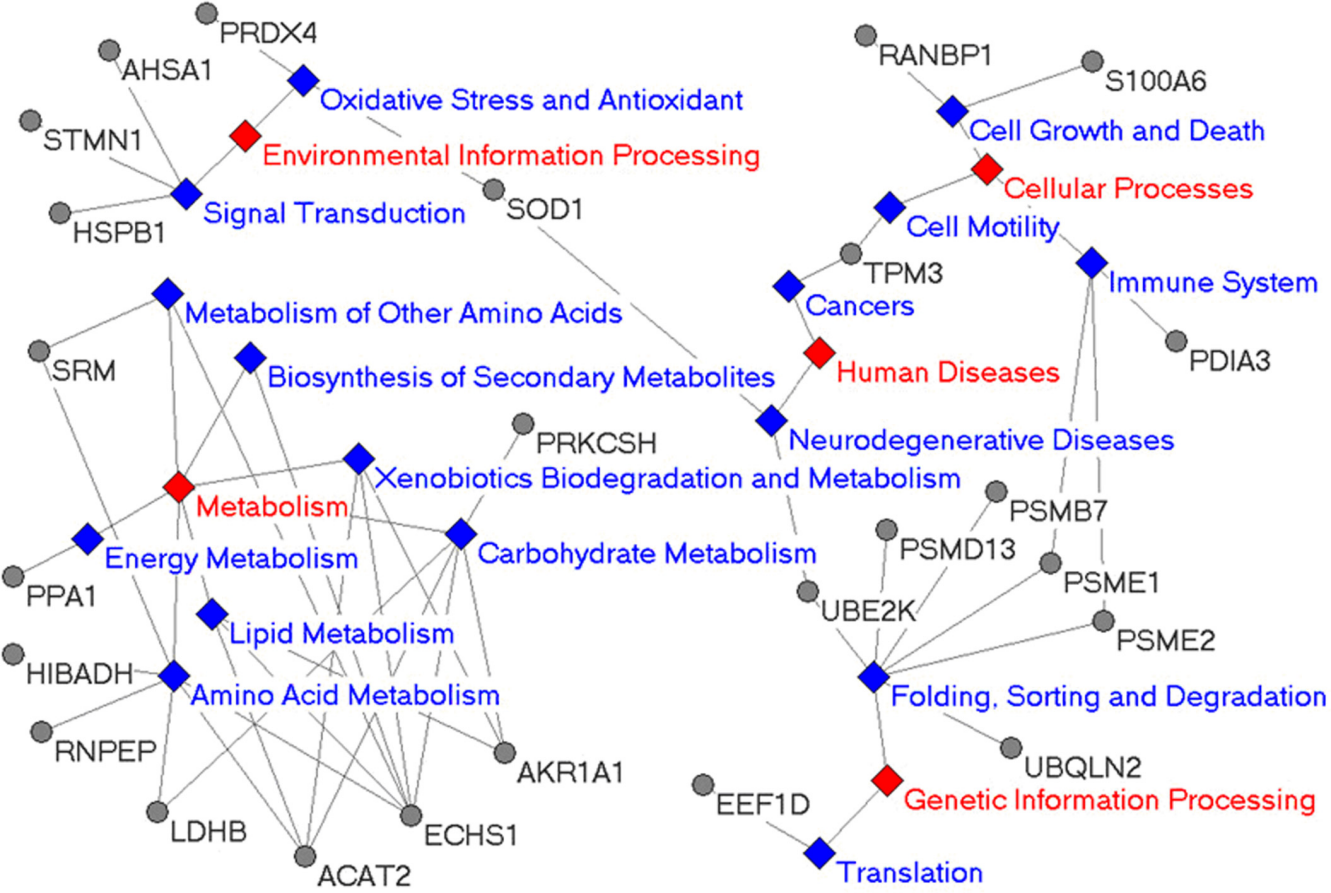
The KEGG pathways related to possible target proteins of arenobufagin (Arg) found in proteomic study. Grey dots were target-related proteins. Full names of these proteins were shown in Table 2. The main pathways were shown in red while the branched pathways were shown in blue.

By mapping KEGG pathway database with possible target-related proteins detected in the proteomic study, the possible pathways related to the cytotoxicity of arenobufagin were found (Fig 3). (i) Unsurprisingly, lots of metabolism pathways including energy metabolism, carbohydrate metabolism, lipid metabolism, amino acid metabolism and others were found to be related to the cytotoxicity of arenobufagin. (ii) Besides, arenobufagin was shown to affect the pathways of cellular processes, such as cell growth and death, cell motility and immune system, and the pathways of human diseases, such as cancer and neuorodegenerative disease. The effect of cardiac steroids/cardiac glycosides on the pathways related to cancer cells was well-known. Interestingly, Piccioni F et al reported that cardiac glycosides might play important roles in the therapy of spinobulbar muscular atrophy and other polyglutamine-related neurodegenerative diseases[42]. (iii) Furthermore, arenobufagin was also found to affect the pathways related to environment information processing, such as oxidative stress pathways and antioxidant pathways. The effect of cardiac steroids/cardiac glycosides on the production of ROS was reported before[43–45]. In the present study, the effect of arenobufagin on the level of cellular ROS and the effect of ROS scavenger on the cytotoxicity of arenobufagin were also checked in the following experiments. (iiii) Most interestingly, 6 differentially expressed proteins were found to be involved in the pathways related to protein folding, sorting and degradation in the processing of genetic information. Furthermore, these 6 proteins were all closely related to the function of proteasome. In brief, the result suggested that arenobufagin might affect the function of proteasome. So, the cellular proteasomal activity of HeLa cells treated with arenobufagin was checked in the following study.

### Arenobufagin directly targeted Na, K-ATPase by computational molecular docking

**Fig 4:**
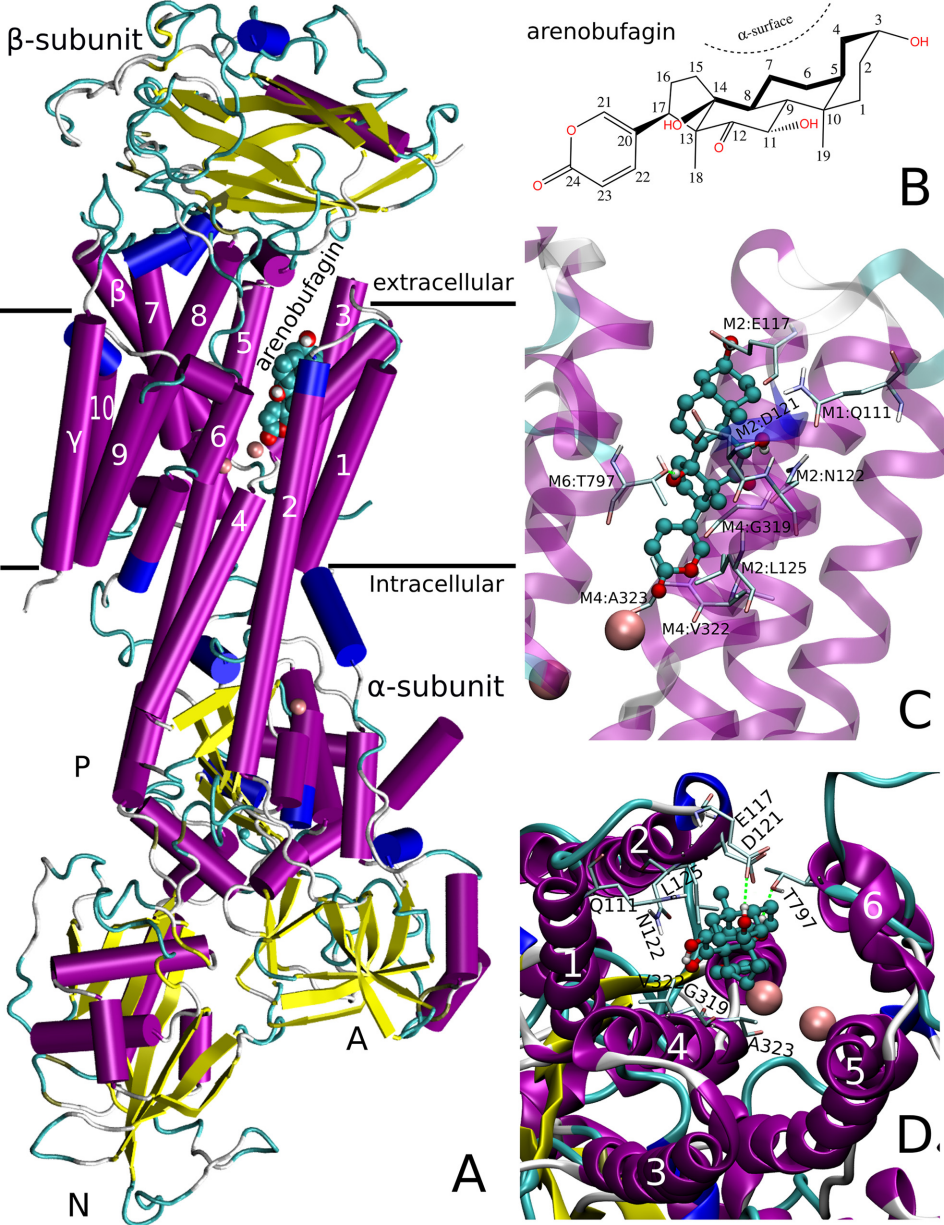
The molecular structures of Na, K-ATPase α_1_β_1_γ E2P-arenobufagin complex and the detailed binding mode were shown by computational molecular docking. (**A)** The molecular structure of Na, K-ATPase α_1_β_1_γ E2P and the binding site of arenobufagin in the Na, K-ATPase α1β1γ E2P. (**B)** The molecular structure of arenobufagin. (**C)** The binding site of arenobufagin visualized from alpha M2 and M4. (**D)** The binding site of arenobufagin visualized from the top of alpha M1-6 and the interaction of arenobufagin and the residues of alpha M1-6. Hydrogen bonds were displayed as green dashed lines and amino acid residues interacted with arenobufagin were also shown.

The active alpha unit sequence of Na, K-ATPase had high identities to alpha isoforms of homo species and other species, which was shown in Table 3. The calculation results of sequence identity for molecular docking simulation indicated that the sequences of Na, K-ATPase alpha 1 subunits with crystal structure in Protein Data Bank (PDB) had high identity to human Na, K-ATPase alpha subunits, which were utilized in the present study. As shown in Fig 4, the results of computational molecular docking revealed that arenobufagin was bound in the cavity formed by the transmembrane alpha subunits M1-6 of Na, K-ATPase, which blocked the pathway of extracellular Na^+^/K^+^ cation exchange and inhibited the function of ion exchange. The molecular structures of Na, K-ATPase α_1_β_1_γ E2P and arenobufagin were displayed in Fig 4A and 4B, respectively. The optimized structure of arenobufagin was docked into the active site of Na^+^/K^+^ ATPase α_1_β_1_γ. The detailed binding mode of arenobufagin and Na, K-ATPase α_1_β_1_γ E2P was shown in Fig 4C and 4D, respectively. Arenobufagin was bound in the extracelluar ion pump port formed by the transmembrane alpha M1-6 subunits, which blocked the Na^+^/K^+^ cation exchange pathway [46, 47]. The ligand arenobufagin was mainly surrounded by the active pocket formed by the residues of Glu117(E117), Asp121(D121), Leu125(L125), Asn122(N122), Gln111(Q111), Thr797(T797), Gly319(G319), Val322(V322), Ala323(A323) and potassium ion in the alpha M1-6 (Fig 4C and 4D). The overall binding mode of Na, K-ATPase α_1_β_1_γ E2P-arenobufagin complex was similar to other cardiotonic steroid such as digoxin and bufalin [46]. The concave side (α-surface) of arenobufagin steroid faced toward hydrophobic side chains of alpha M4-6, which served as a general docking site for the cardiotonic steroids [46, 47]. Arenobufagin interacted with polar side chain of alpha M1-2. OH_3_ and OH_14_ of arenobufagin formed the hydrogen bonding with Glu117 (αM6) and Thr797 (αM1-2), respectively (Fig 4C and 4D). The lactone ring of arenobufagin was extended into a deep hydrophobic channel formed by alpha M4-6, which might be dragged toward the cation-binding site by K^+^.

**Table 3:** Sequence identity between human alpha subunits and alpha 1 of other species (PDB ID: 4RES, 4HYT and 4XE5).

Subunit	4RES	4HYT	4XE5
	Identity (%)	Identity (%)	Identity (%)
Human α_1_	98.24	98.14	97.55
Human α_2_	86.57	86.57	86.67
Human α_3_	87.50	87.50	87.69
Human α_4_	78.69	78.69	78.49

### Arenobufagin decreased Na, K-ATPase activity and increased the level of cellular Ca(2+) and ROS

**Fig 5:**
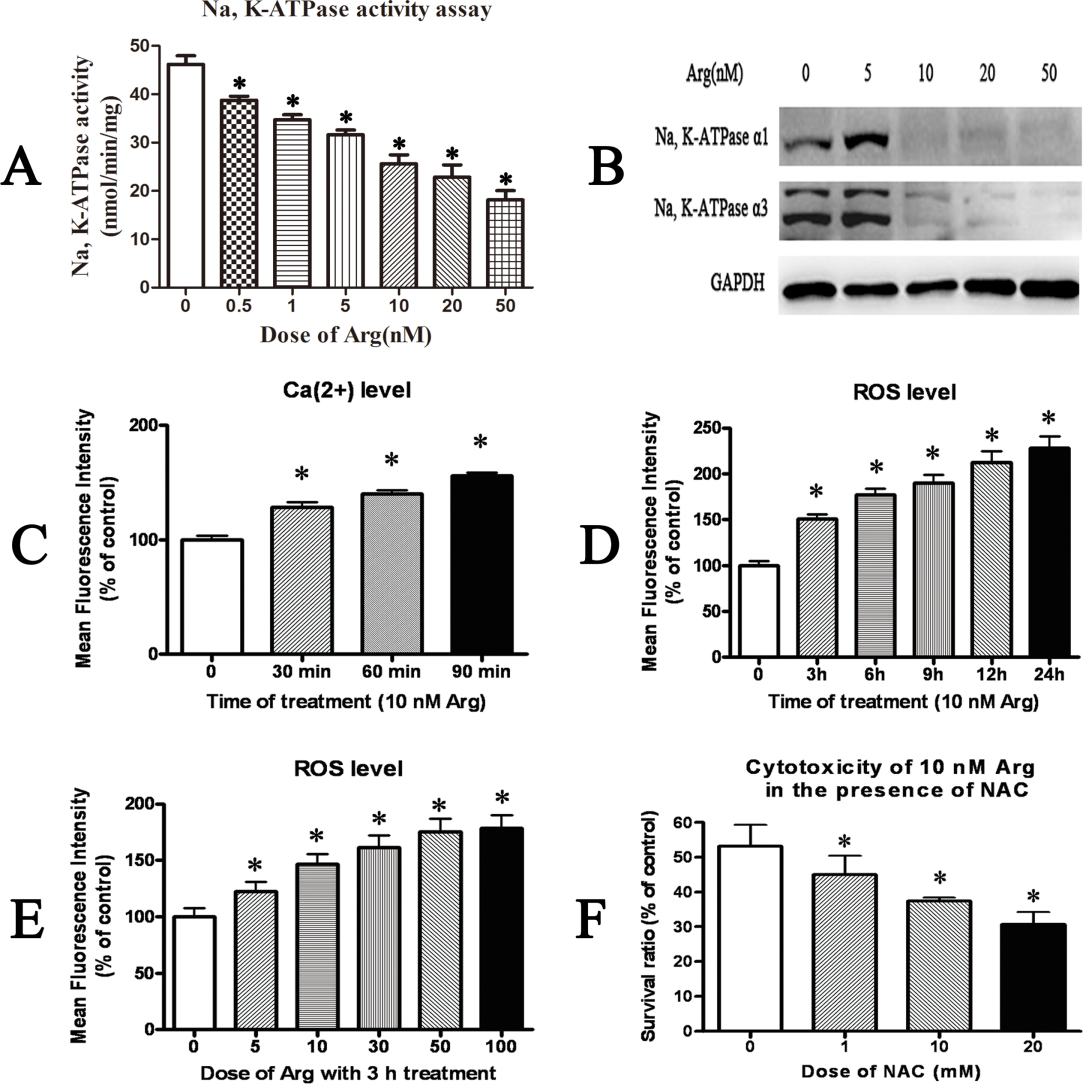
Effect of arenobufagin (Arg) on Na, K-ATPase activity, intracellular Ca(2+) level and ROS level. (**A**) Quantitation of Na, K-ATPase activity in HeLa cells treated with arenobufagin(Arg) at different doses for 24h. (B) Western blotting results of Na, K-ATPase α1 and α3 in HeLa cells treated with arenobufagin(Arg) at different doses for 24 h. (**C**) Quantitation of the intracellular Ca(2+) level in HeLa cells treated with 10 nM arenobufagin for different time periods. (**D**) Quantitation of the intracellular ROS level in HeLa cells treated with 10 nM arenobufagin for different time periods. (**E**) Quantitation of the intracellular ROS level in HeLa cells treated with arenobufagin at different doses for 3h. (F) The viability of HeLa cells treated with 10 nM arenobufagin for 72 h in the presence of ROS scavenger NAC (N-acetyl cysteine). Data were statistical results of three independent experiments. Data were expressed as mean ± SD. *Significant difference from the control group at P<0.05.

Similar to previous studies[29, 35, 36], the involvement of Na, K-ATPase, Ca2+, reactive oxygen species (ROS) and anti-oxidant proteins related to the cytotoxicity of arenobufagin was also found in our study. As shown in Fig 5A, Na, K-ATPase activity of HeLa cells treated with arenobufagin at different doses (0.5nM, 1nM, 5nM, 10nM, 20nM, 50nM) for 24h was significantly inhibited. And, the expression of Na, K-ATPase α1 and α3 subunits was also decreased by arenobufagin in HeLa cells, respectively (Fig 5B). As shown in Fig 5C, the level of cellular Ca (2+) was significantly increased in HeLa cells treated with arenobufagin for 30 min. And, the level of cellular ROS was increased by arenobufagin time-dependently (Fig 5D) and dose-dependently (Fig 5E). The cytotoxicity of arenobufagin could be inhibited in HeLa cells pretreated with ROS scavenger NAC (Fig 5F). In the presence of NAC, the viability of HeLa cells treated with arenobufagin was significantly increased.

### Arenobufagin inhibited cellular proteasomal activity, which might be related to the binding of Na, K-ATPase

**Fig 6:**
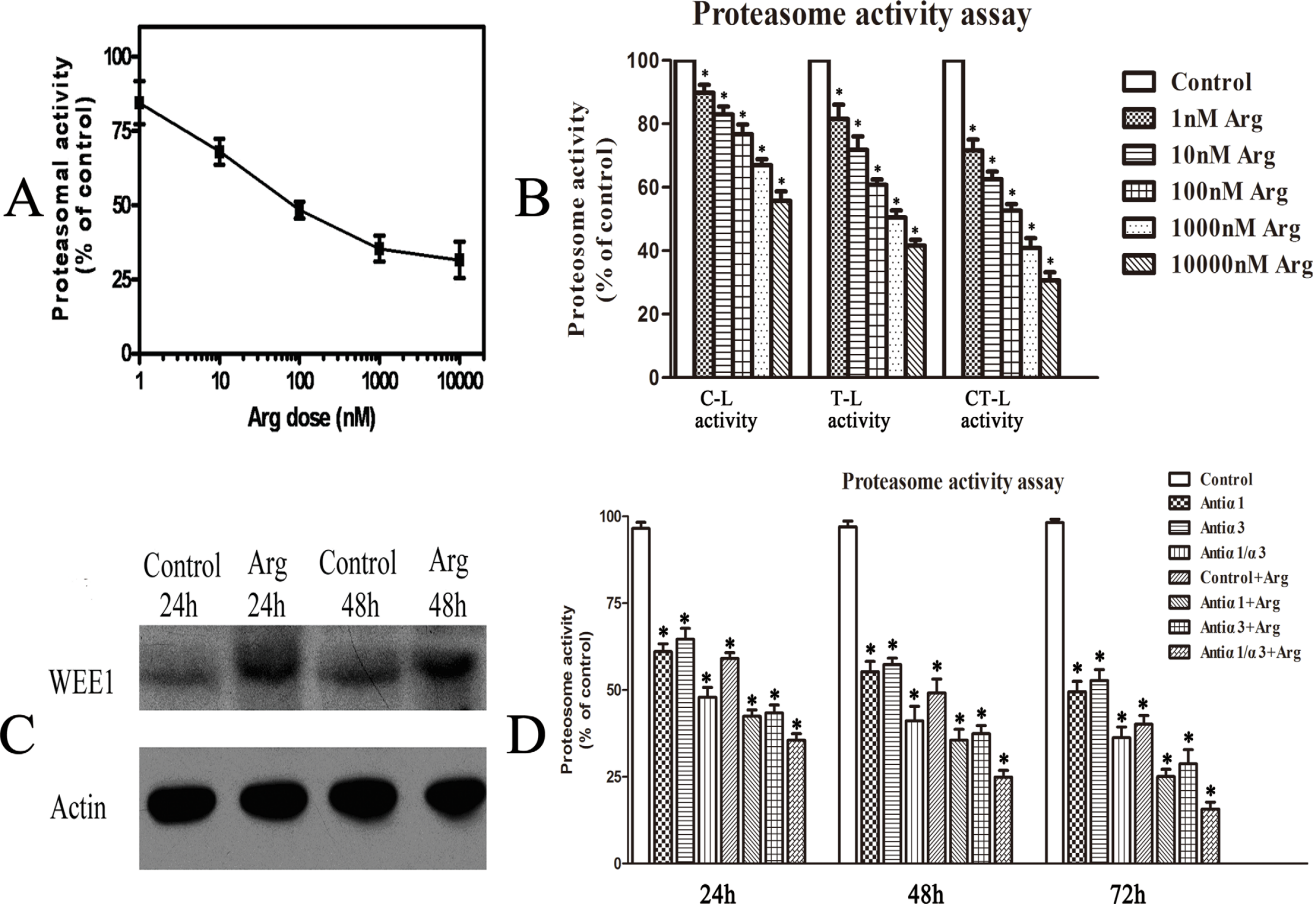
Cellular proteasomal activity was inhibited by arenobufagin, which might be related to the binding of Na, K-ATPase. **(A**) Inhibition of arenobufagin (Arg) at different doses on cytosolic proteasomal activity for 24h. (**B**) Three types of cellular proteasome enzyme activities (C-L, T-L and CT-L) in HeLa cells treated with 0.1% DMSO or arenobufagin(Arg) at different concentrations for 24h. (**C**) Western blotting results of WEE1 and actin in control HeLa cells and cells treated with 10 nM arenobufagin for 24 h and 48 h. Each blot was the representative result of three independent experiments. (**D**) Inhibition of antibodies against α1 or α3 subunits of Na,K-ATPase or combination of antibodies against α1 or α3 subunits of Na,K-ATPase and 10 nM arenobufagin on cytosolic proteasomal activity for 24h,48h and 72h, respectively. Data were statistical results of three independent experiments. Data were statistical results of three independent experiments. Data were expressed as mean ± SD. *Significant difference from the control group at P<0.05.

More interestingly, our proteomic result indicated that the change of proteasome function might be involved in the cytotoxicity of arenobufagin. As shown in Fig 6A, cellular proteasomal activity in HeLa cells could be dose-dependently decreased by arenobufagin. The IC_50_ value of arenobufagin on cellular proteasomal activity for 24h was calculated to be 242.12 ± 104.21 nM. Proteasome β1, β2 and β5 subunits exert caspase-like (C-L), trypsin-like (T-L) and chymotrypsin-like (CT-L) activity, respectively. As shown in Fig 6B, arenobufagin could inhibit all three types of cellular proteasome enzyme activities (C-L, T-L and CT-L) in HeLa cells for 24h. And, the expression of WEE1 was increased in HeLa cells treated with arenobufagin (Fig 6C). WEE1 is an inhibitory kinase of cyclin-dependent kinase and therefore it plays important roles in the control of cell cycle. WEE1 is down-regulated primarily through proteasome-dependent degradation after ubiquitylated by the E3 ubiquitin ligase[48,49]. Furthermore, to check whether the inhibitive effect of arenobufagin on proteasomal activity was related to its binding with Na, K-ATPase, the effects of antibodies against Na, K-ATPase α1 and α3 subunits alone or combinated with 10 nM arenobufagin for 24h, 48h and 72h were also checked, respectively. The result that cellular proteasomal activity in HeLa cells could be inhibited by antibodies against antibodies against Na, K-ATPase α1 and α3 subunits alone or combinated with 10 nM arenobufagin was shown in Fig 6D. Compared with antibodies against Na, K-ATPase α1 and α3 subunits alone, the inhibitive proteasomal activity of HeLa cells was significantly changed by antibodies against Na, K-ATPase α1 and α3 subunits combinated with 10 nM arenobufagin for 24h, 48h and 72h, respectively. These results indicated that arenobufagin might directly bind with Na, K-ATPase α1 and α3 subunits and the inhibitive effect of arenobufagin on proteasomal activity of HeLa cells might be related to its binding with Na, K-ATPase.

### Ataxin-1 and translationally-controlled tumor protein might be intermediate proteins between Na, K-ATPase and proteasome

**Fig 7:**
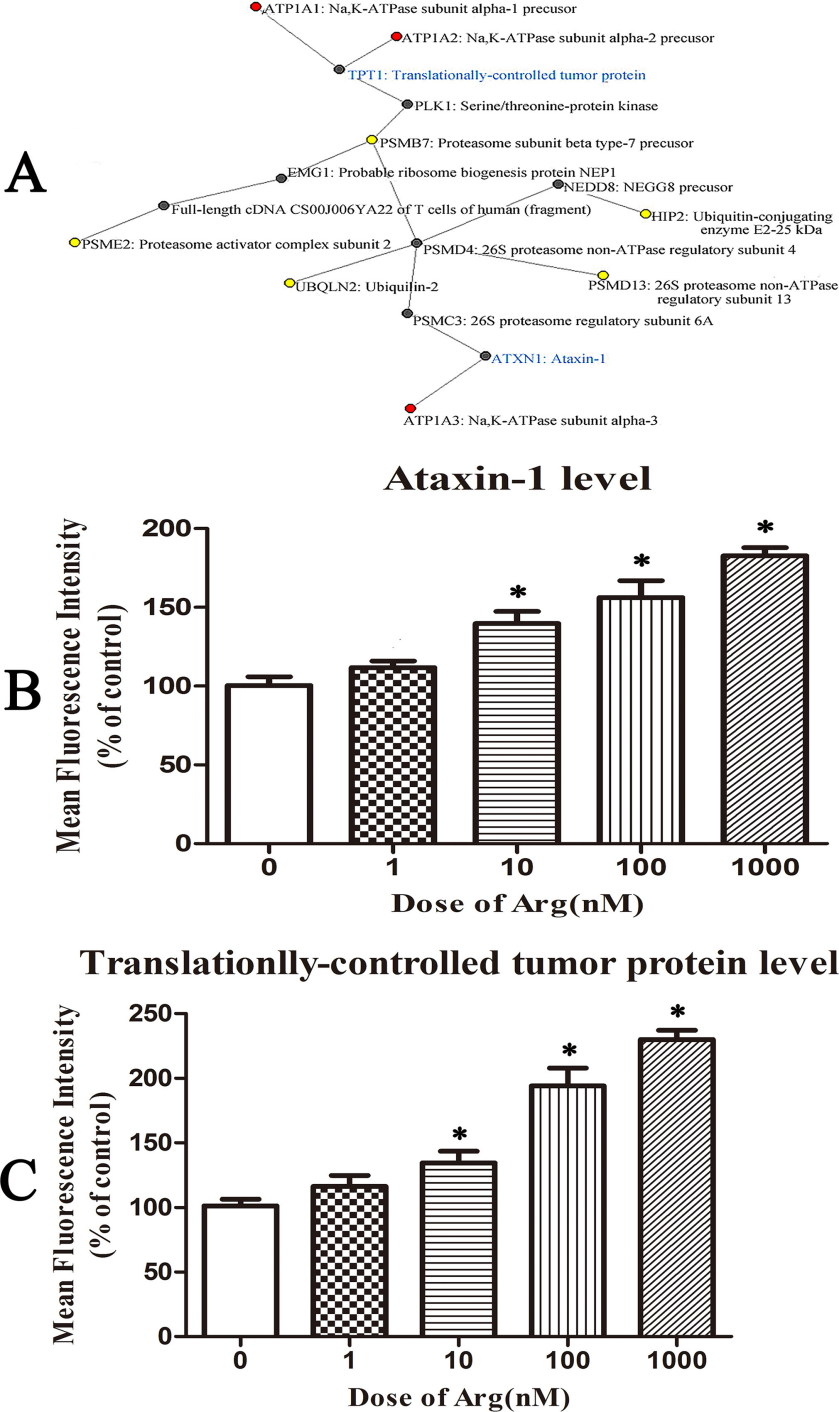
Ataxin-1 and translationally-controlled tumor protein might be intermediate proteins between Na, K-ATPase and proteasome. (**A**) Protein-protein interaction network constructed to connect α1, α2 or α3 subunits of Na,K-ATPase and proteasomal-related proteins found in the proteomic study. The red dots were α1, α2 or α3 subunits of Na,K-ATPase and the yellow dots were 5 proteasomal-related proteins found in the proteomic study. The intermediate partner proteins except ataxin-1 and translationally-controlled tumor protein were shown in black. Ataxin-1 and translationally-controlled tumor protein (shown in blue) were considered as the most important intermediate partners between Na,K-ATPase and proteasome. (**B**) The protein expression level of ataxin-1 in control HeLa cells or cells treated with arenobufagin at different doses for 24 h. (**C**) The protein expression level of translationally-controlled tumor protein in control HeLa cells or cells treated with arenobufagin at different doses for 24 h. Data were statistical results of three independent experiments. Data were expressed as mean ± SD. *Significant difference from the control group at P<0.05.

Ataxin-1 is an ubiquitous protein well conserved in vertebrates, which is typically expressed in nuclear of neurons[50].Ataxin-1 plays an important role in protecting against aggregation of neurodegenerative spinocerebellar ataxia type 1[51]. Translationally controlled tumor protein (TCTP) is a highly conserved protein, which is widely expressed in the cytoplasmic and the nucleus of all eukaryotic organisms[52]. The expression of TCTP is highly regulated by various extracellular stimuli, both at the transcriptional and translational level. TCTP has been shown to play an important role in physiological events, such as cell proliferation, cell growth, cell cycle progression, cell death, immune responses, chemoresistance, malignant transformation and and tumor reversion[53]. As shown in Fig 7A, the possible signal cascade between Na,K-ATPase and proteasome-related proteins found in the present study was predicted based on protein-protein interaction database by bioinformatic analysis. Among 6 proteasome-related proteins found in the present study, 5 of them could be found to be linked to Na,K-ATPase through only two or three intermediate proteins in the possible signal cascade (Fig 7A). Ataxin-1 and translationally-controlled tumor protein might be important intermediate proteins between Na,K-ATPase and proteasome in the protein-protein interaction network. Therefore, the expression levels of ataxin-1 and translationally-controlled tumor protein in HeLa cells treated with arenobufagin were checked. As shown in Fig 7B and 7C, the expression levels of ataxin-1 and translationally-controlled tumor protein could be significantly increased in HeLa cells treated with arenobufagin at the dose of 10 nM, 100nM or 1000 nM for 24 h, respectively.

## Discussion

Arenobufagin, extracted from toad venom (traditional Chinese medicine “Chan-Su”) is a major bufadienolide as a candidate anti-cancer drug. Until now, biological activities and anti-cancer mechanisms of arenobufagin aren’t still fully clear and only a few researches have been carried out. In the present study, arenobufagin was shown to be cytotoxic against HeLa cells. Arenobufagin could effectively inhibit the growth of HeLa cells. In addition, the results of flow cytometry assay indicated that arenobufagin might induce cell cycle arrest at G2/M phase and trigger apoptosis of HeLa cells. Our results were consistent with previous reports about the cytotoxicity of bufalin, which structure was similar with the structure of arenobufagin. For example, bufalin induced G2/M phase arrest and apoptosis of human osteosarcoma cells, leukemia cells and endothelial cells[54–56]. Consistent with previous reports on the basis of the partitioning of a lipophilic cationic fluorescent dye rhodamine 123[57], our flow cytometry analysis also indicated that the plasma membrane potential of HeLa cells was dose-dependently decreased by treatment with arenobufagin for 3h.

Since the signal cascade pathways affected by cardiac steroids/cardiac glycosides are not fully clear, a proteomic method is used to globally search for the possible target-related proteins as well as signal-related pathways in the present study. Proteomic technology has become an indispensable and efficient tool in biochemical research. This technology is particularly suitable to study the mechanism of compounds like cardiac steroids/cardiac glycosides, which could affect lots of target-related proteins and complicated signal cascades. 25 possible target-related proteins of arenobufagin were successfully detected by proteomic assay in the present study. After mapping these possible target-related proteins in KEGG pathway database by bioinformational assay, we found that arenobufagin might affect the pathways including metabolism, oxidative stress and antioxidant proteins, cell growth and death, cell motility, protein folding, sorting and degradation, and others. The involvement of the pathways, which were related to metabolism, oxidative stress, cell motility as well as cell growth and death, was in accordance to previous reports. For example, cardiac steroids/cardiac glycosides had been known to inhibit aerobic glycolysis [58] and Lopez-Lazaro M reported that inhibition of Na, K-ATPase and concomitant inhibition of glycolysis might explain the anticancer effects of cardiac glycosides[59]. Ionic homeostasis including the levels of Na(+), K(+) and Ca(2+) maintained by Na, K-ATPase ion pump was critical for cell growth, cell differentiation, cell survival and cell migration[60–62]. The change of intracellular Na(+), K(+) and Ca(2+) levels could be caused by the binding of cardiac steroids/cardiac glycosides to Na, K-ATPase.

Serving as a multifunctional integrator and signal transducer, Na, K-ATPase is responsible for maintaining resting potential and regulating cellular volume, contractility, inflammation, adhesion and apoptosis [63, 64]. The aberrant activity and expression of Na, K-ATPase have been implied in the progression of several types of aggressive cancers. Na, K-ATPase α1 and α3 subunits are frequently overexpressed in colorectal cancer, non-small cell lung cancer, melanomas and glioblastomas [65–67]. In the present study, the results of computational molecular docking revealed that arenobufagin was bound in the cavity formed by the transmembrane alpha subunit of Na, K-ATPase, which blocked the pathway of extracellular Na^+^/K^+^ cation exchange and inhibited the function of ion exchange. Since a decrease in the plasma membrane potential reflected the inhibition of Na, K-ATPase on the plasma membrane of cells[57], our findings indicated that arenobufagin inhibited Na, K-ATPase activity of HeLa cells and the expression of Na, K-ATPase α1 and α3 subunits in HeLa cells treated with arenobufagin was significantly decreased. The inhibition of Na, K-ATPase leads Na+ and Ca(2+) ions to accumulate in tumour cells as well as reduce the membrane potential and the intracellular levels of K+ [68]. Intracellular Ca (2+) overload can lead to cell death because Ca(2+) participates in a variety of cell death programs [69]. Our findings also certified that intracellular Ca(2+) level in HeLa cells treated with arenobufagin in the present study. Furthermore, the increase of ROS level by cardiac steroids/cardiac glycosides in cardiac myocytes as well as in cancer cells has been reported[43,44]. In the present study, we also observed that the level of intracellular ROS in HeLa cells treated with arenobufagin was increased, which supported the result of pathway analysis by bioinformational assay in our study. The inhibition of ROS scavenger on the cytotoxicity of arenobufagin indicated that the increase of ROS could partly contribute to the cytotoxicity of arenobufagin to HeLa cells.

To be noted, the most interesting finding in the results of our proteomic and bioinformational analysis was the possible involvement of proteasome, which participated in the pathways related to protein folding, sorting and degradation pathway, in the cytotoxicity of arenobufagin to HeLa cells. Six target-related proteins of arenobufagin, i.e. ubiquilin 2, proteasome beta 7 subunit protein, proteasome activator subunit 1 isoform 1, proteasome activator complex subunit 2, proteasome 26S non-ATPase subunit 13 isoform 1 and ubiquitin-conjugating enzyme E2-25 kDa, were proteasome-related proteins in the present study. Ubiquilin 2 was also called as the protein linking IAP with cytoskeleton 2, which functioned in targeting ubiquitinated proteins to the proteasome[70,71]. Proteasome beta 7 subunit protein, proteasome activator subunit 1 isoform 1, proteasome activator complex subunit 2, proteasome 26S non-ATPase subunit 13 isoform 1 and ubiquitin-conjugating enzyme E2-25 kDa were all directly involved in the function of proteasome[72]. Based on these results, we predicted that the function of proteasome in HeLa cells was affected by arenobufagin. To certify the prediction, the effect of arenoibufagin on the activity of cellular proteasome was checked in the present study. The result indicated that proteasomal activity could be inhibited in HeLa cells treated with arenobufagin. Arenobufagin could dose-dependently inhibit caspase-like (C-L), trypsin-like (T-L) and chymotrypsin-like (CT-L) activity, mediated by proteasome β1, β2 and β5 subunits, respectively. The protein level of WEE1, which could be degraded by proteasome, was increased in HeLa cells treated with arenobufagin. Since WEE1 played an important role in the control of cell cycle, the increase of WEE1 level might contribute to G2/M phase arrest of HeLa cells induced by arenobufagin. Besides, the activities of proteasome in HeLa cells treated with bufalin and digitoxin were also shown similar effects with arenobufagin, respectively (data not shown).

In the present study, the finding that the functions of proteasome and proteasome-related proteins were affected by arenobufagin shed new light on the anti-cancer mechanism of arenobufagin as well as cardiac steroids/cardiac glycosides. Firstly, the proteasomal activity inhibited by arenobufagin might partly contribute to the cytotoxicity of arenobufagin to HeLa cells. It was well known that the proteasome was essential for the survival of cancer cells and proteasome inbititors could exhibit anti-cancer activity by inducing growth arrest and/or apoptosis of cancer cells[73,74].Ubiquilin 2, which was found in the present study as one of possible target-related proteins of arenobufagin and belonged to the proteasome-related protein, was reported to be involved in endocytosis[75]. Interestingly, Na, K-ATPase and cardiac steroids/cardiac glycosides were both shown to play important roles in the regulation of endocytosis[76]. Secondly, the function of proteasome inhibited by arenobufagin might also partly contribute to the reported effect of cardiac steroids/cardiac glycosides on neurodegenerative diseases. For example, ubiquitin-conjugating enzyme E2-25 kDa, which was found in the present study as one of possible target-related proteins of arenobufagin, was reported to be involved in the aggregate formation and cell death connected with polyglutamine diseases[77]. To further check whether the effect of arenobufagin on proteasome was related to Na, K-ATPase binding or not, the proteasomal activities of HeLa cells treated with antibodies against Na, K-ATPase α1 and α3 subunits alone or combinated with arenobufagin were also examined in our study. In the present study, proteasomal activity of HeLa cells could be inhibited by antibodies against Na, K-ATPase alone or combinated with arenobufagin. Arenobufagin was bound in the cavity formed by the transmembrane alpha subunit (α1 and α3) of Na, K-ATPase by computational molecular docking and antibodies against Na, K-ATPase α1 and α3 subunits was bound in the N-terminus of alpha subunit (α1 and α3) of Na, K-ATPase, according to antibody manuals. Compared with antibodies against Na, K-ATPase α1 and α3 subunits alone, the inhibitive proteasomal activity of HeLa cells was significantly changed by antibodies against Na, K-ATPase α1 and α3 subunits combinated with arenobufagin. These results indicated that arenobufagin might directly bind with Na, K-ATPase α1 and α3 subunits and the inhibitive effect of arenobufagin on proteasomal activity of HeLa cells might be related to its binding with Na, K-ATPase.

In all, by the methods of proteomic and bioinformational analysis, a new signal cascade pathway affected by arenobufagin was found in the present study. The inhibitive effect of arenobufagin on proteasomal activity might be based on its binding with Na, K-ATPase in HeLa cells. The effects of cardiac steroids/cardiac glycosides on proteasomal activity by binding with Na, K-ATPase had been reported before. Zhang D et al first indicated ouabain modulated proteasome-mediated proteolysis of AMPA receptor (AMPAR) by directly binding to Na,K-ATPase[78]. Moreover, proteasome-related proteins /genes had been reported to be involved in the effects of cardiac steroids/cardiac glycosides. For example, Chen A et al reported ubiquitin-conjugating enzyme E2M (UBC12 homolog, yeast) gene was found to be one of the differentially expressed genes in HL-60 cells treated with bufalin, another bufadienolide isolated from toad venom[79]. Qiu J et al reported proteasome subunit 5 was found to be possible target-related protein of digoxin in endothelial cells by proteomic assay[80]. Our results were consistent with these reports. While, our results of bioinformational analysis and preliminary experiments suggested that ataxin-1 and translationally-controlled tumor protein might be intermediate partner proteins between Na, K-ATPase and proteasome in HeLa cells treated with arenobufagin. Further work is needed to certify the role of cardiac steroids/cardiac glycosides as well as Na, K-ATPase in the function of proteasome and characterize detailed signal cascade pathways from Na, K-ATPase to proteasome.
